# Pan-Inhibitory Hippocampal Neuron Ablation Reveals Insights into the Role of Interneurons in Epileptogenesis

**DOI:** 10.1523/ENEURO.0229-24.2024

**Published:** 2024-07-03

**Authors:** Chandni Rana, Joanna Mattis

**Affiliations:** ^1^ Neuroscience Graduate Program, University of Michigan, Ann Arbor, Michigan 48104; ^2^ Department of Neurology, University of Michigan, Ann Arbor, Michigan 48104; ^3^ Michigan Neuroscience Institute, University of Michigan, Ann Arbor, Michigan 48104

Impaired inhibition—and the resulting disruption of the brain's excitatory–inhibitory (E–I) balance—has historically been theorized to play a critical role in epileptogenesis and seizures (Treiman, 2001). This concept is supported by numerous studies showing impaired function of gamma-aminobutyric acid (GABA)-ergic function in both genetic and acquired animal models of epilepsy and in human epileptic brain tissue. Furthermore, medications that enhance GABAergic signaling have an anticonvulsant effect whereas GABA antagonists are proconvulsant.

Yet, despite a large body of evidence implicating GABA in epileptogenesis and epilepsy, the direct causal role of inhibitory interneuron loss (or impairment) has remained incompletely understood. In the case of acquired models of epilepsy, such as temporal lobe epilepsy, interneuron loss is central to signature pathophysiology but occurs alongside multiple other pathological and neuroplastic changes that are also likely to influence disease progression. In the case of genetic epilepsies, such as Dravet syndrome, interneurons display prominent pathology (Ogiwara et al., 2007), but developmental abnormalities likewise complicate interpretation. Several studies have attempted to selectively inhibit interneurons in the adult hippocampus to isolate their effects on epileptogenesis, but these manipulations were limited to specific inhibitory cell subpopulations (Drexel et al., 2017) or hippocampal subregions (Spampanato and Dudek, 2017) and the results were modest. Thus, definitively implicating inhibitory interneuron loss has remained an elusive goal.

A recent study by Dusing et al. (2024) aimed to answer this longstanding question of how interneuron loss—in isolation of other factors—contributes to epileptogenesis and epilepsy. Their innovative experimental approach employed inducible, pan-inhibitory neuron ablation or silencing, targeted widely across the hippocampus. Specifically, the authors first used diphtheria toxin-mediated ablation targeted to vesicular GABA transporter (VGAT)-expressing interneurons in the dorsal and ventral bilateral hippocampus. This led to the development of frequent and severe seizure clusters and persistent epileptiform activity as measured by EEG. Surprisingly, 1 week later, there was a dramatic decline in seizure frequency, with only occasional breakthrough seizures thereafter. Postexperimental immunostaining showed significant reductions in the density of parvalbumin (PV) and somatostatin (SST) immunoreactive interneurons across the dorsal and ventral hippocampus, supporting the assumption that most inhibitory interneurons were indeed ablated during the experiment. Interestingly, upon closer examination of perisomatic inhibitory inputs onto dentate gyrus granule cells (GCs), while the number of PV puncta was markedly reduced (consistent with the decrease in PV interneuron number), the density of postsynaptic inhibitory synapses (assayed by immunostaining for gephyrin, a postsynaptic scaffolding protein at inhibitory synapses) was unchanged. Next, the authors conducted transient silencing of the same VGAT neuronal population by activating the chemogenetic inhibitory receptor, hM4DGi, expressed within those cells. Chemogenetic inhibition of VGAT neurons led to interictal spikes and seizures, though the observed phenotype was significantly milder than had been observed with interneuron ablation.

The findings presented by Dusing et al. (2024) both answer and raise questions about inhibition in epileptogenesis and epilepsy. First, as expected, targeted ablation or silencing of inhibitory neurons did indeed trigger seizures. However, approximately half of the mice were observed to have spontaneous seizures at baseline (i.e., beyond the acute postsurgical time window, but prior to experimental interneuron manipulation), likely reflecting disruption of the endogenous *Vgat* gene in the transgenic line used for the studies, so these data unfortunately cannot be used to unequivocally conclude that interneuron loss is sufficient to cause epilepsy. It also remains unclear whether the difference in seizure severity between both types of manipulations merely reflected a difference in magnitude of loss of inhibition or whether the acute (silencing) versus subacute (ablation) timescales of the experiment may have contributed.

Second, despite a robust and persistent decline in inhibitory cell number, hippocampal circuitry demonstrated a surprising capacity to regain homeostasis, as evidenced by the dramatic decline in seizure activity ∼1 week following interneuron ablation. What may account for this observation? The authors found a preservation of postsynaptic GABA receptors on dentate gyrus GCs, but this data was only collected from a single timepoint: the terminal end of the experiment (i.e., after relative homeostasis had been regained). It would be illuminating to see whether postsynaptic GABA receptors are similarly maintained at a “nonhomeostatic” timepoint corresponding with peak seizure burden. Furthermore, because postsynaptic inhibitory synapses were maintained despite reduced PV puncta, another pressing inquiry is to determine the source of this inhibition. One possibility is that surviving PV interneurons undergo sprouting and also downregulate the parvalbumin protein to levels undetectable with immunostaining. A second possibility is sprouting from surviving PV-negative interneurons within the dentate gyrus. A third possibility is an increase in long-range inhibitory input, for example, from the medial septum. Finally, a fourth possibility is a more dramatic reorganization of the local circuit, in which dentate gyrus GCs become a paradoxical source of local inhibitory input. A series of electrophysiology studies by Gutiérrez provides theoretical support for this final possibility: simultaneous glutamatergic and GABAergic transmission from GC mossy fibers was observed after seizures, concurrent with an overexpression of granule cell proteins involved in GABA synthesis (Gutiérrez, 2000). Any of these could allow the circuit to regain homeostasis following the initial loss of inhibition, and future experiments could be designed to determine which is the dominant process.

Finally, and related to the above, although Dusing et al. (2024) achieved a robust finding with broad, pan-inhibitory ablation, it is well established that different interneuron subtypes play distinct roles in seizures. How are the different interneuron subtypes engaged throughout the course of ablation-induced seizures and the eventual return to homeostasis? Further study into these questions will be vital to advance our understanding and treatment of epileptogenesis and epilepsy.
